# An atlas of posttranslational modifications on RNA binding proteins

**DOI:** 10.1093/nar/gkac243

**Published:** 2022-04-19

**Authors:** Whitney E England, Jingtian Wang, Siwei Chen, Pierre Baldi, Ryan A Flynn, Robert C Spitale

**Affiliations:** Department of Pharmaceutical Sciences, University of California, Irvine. Irvine, CA, USA; Department of Pharmaceutical Sciences, University of California, Irvine. Irvine, CA, USA; School of Information and Computer Sciences, University of California, Irvine. Irvine, CA, USA; School of Information and Computer Sciences, University of California, Irvine. Irvine, CA, USA; Stem Cell Program, Boston Children's Hospital, Boston, MA, USA; Department of Stem Cell and Regenerative Biology, Harvard University, Cambridge, MA, USA; Department of Pharmaceutical Sciences, University of California, Irvine. Irvine, CA, USA; Department of Developmental and Cellular Biology, University of California, Irvine. Irvine, CA, USA; Department of Chemistry, University of California, Irvine. Irvine, CA, USA

## Abstract

RNA structure and function are intimately tied to RNA binding protein recognition and regulation. Posttranslational modifications are chemical modifications which can control protein biology. The role of PTMs in the regulation RBPs is not well understood, in part due to a lacking analysis of PTM deposition on RBPs. Herein, we present an analysis of posttranslational modifications (PTMs) on RNA binding proteins (RBPs; a PTM RBP Atlas). We curate published datasets and primary literature to understand the landscape of PTMs and use protein–protein interaction data to understand and potentially provide a framework for understanding which enzymes are controlling PTM deposition and removal on the RBP landscape. Intersection of our data with The Cancer Genome Atlas also provides researchers understanding of mutations that would alter PTM deposition. Additional characterization of the RNA–protein interface provided from in-cell UV crosslinking experiments provides a framework for hypotheses about which PTMs could be regulating RNA binding and thus RBP function. Finally, we provide an online database for our data that is easy to use for the community. It is our hope our efforts will provide researchers will an invaluable tool to test the function of PTMs controlling RBP function and thus RNA biology.

## INTRODUCTION

RNA molecules have emerged as critical players in nearly every biological process, from cell division to the regulation of chromatin state and gene expression (1,2). Many diseases, ranging from cancer (3,4) to neurological disorders (5), can be traced back to faulty RNA structure, function, or posttranscriptional regulation. Signaling hubs inside cells can also be regulated by RNA molecules, further suggesting more dynamic processes can utilize RNA-centered signaling to control specific outputs to regulate cell fate and disease onset (6). As such, understanding the molecular mechanisms utilized by cells to control RNA expression and fate are critical to creating a complete picture of how RNA molecules are controlled to impart their many biological outputs.

During their lifetimes, RNA molecules are constantly in contact with RNA-binding proteins (RBPs) (7,8). RNA-RBP interactions control and regulate alternative splicing, RNA export and RNA decay. Our expanded understanding of the catalog of such interactions suggests each of these processes can be dynamic with the catalog of RBP–RNA interactions changing to toggle RNA expression for desired cellular output (9–11). Almost all characterization of RBP–RNA interactions has been focused on identifying the protein–RNA interface and have limited focus on how the RBPs themselves are regulated to control RNA fate (12). There is much work to be done to fully understand how RBPs select their RNA targets in cells, how signaling networks can control RBP selection, how RBPs themselves are regulated, and how such regulation can lead to altered RNA expression.

Protein molecules are subject to a high level of regulation through chemical modifications termed posttranslational modifications (PTMs) (13). PTMs are known to regulate protein structure, localization, decay and signaling activities (14,15). PTMs vary widely in their chemical nature and can impart unique structural and charge differences associated with their deposition and removal. Further, proteins with evidence of multiple PTMs have been suggested to be more involved in disease biology than poorly modified proteins (16). PTM marks are dynamic and can either be deposited through enzyme-regulated processes or changes in cellular chemistry (i.e. oxidation). While PTMs have been studied for quite some time through the lens of protein regulation, our understanding and characterization of RBP PTMs is quite limited with only a few examples well characterized to date.

There has been some important headway in characterizing PTM modifications and their role in regulating RNA binding proteins. The limited cases illustrate just how impactful changes to the chemical composition of RBPs can be (17). For example, in the interferon (IFN)-γ-activated inhibitor of translation (GAIT) system(18,19), IFN-γ induces formation of a multiprotein GAIT complex that binds structural GAIT elements in the 3' untranslated regions (UTR) of multiple inflammation-related mRNAs. This complex is recruited and regulated by IFN-γ-induced kinases that phosphorylate RNA binding proteins to control mRNA translation; PTM regulation of RBPs in this case are critical for regulating mRNA interactions and thus the IFN-γ translation response. In another example, there is growing evidence that PTMs can regulate phase separation (20). In the context of tyrosine phosphorylation, it has been shown that tyrosine residues in RBPs can have weak interactions driving phase separation; however, once tyrosine residues are phosphorylated phase separation breaks down due to coulombic repulsion between the negatively charged residues. Overall, such examples highlight the importance of PTM regulation for RBPs, but likely represent only a small fraction of the total examples.

Expanding the suite of known and addressable of PTMs on RBPs requires rigorous curation and interrogation of existing proteome-scale mass spectrometry data coupled to other facets of RNA–protein interaction biology; together this could serve as a platform for novel hypotheses related to functional types and sites of PTMs on RBPs. Herein, we perform a thorough analysis of experimentally derived PTMs. Using recent large-scale datasets for the identification of RNA binding proteins, we investigate PTMs on the set of known RBPs (RBP-ome). We intersect this dataset with published protein–protein interaction databases to provide potential models for how PTMs are deposited and removed by cellular enzymes. We utilize mass spectrometry data on in-cell crosslinked RNA–protein interactions, that are close to identified RNA–protein interfaces, to provide the community with PTMs that have the potential to regulate RNA–protein binding. We investigate the potential disease relevance of PTM sites by intersecting our data with The Cancer Genome Atlas. We catalog novel PTM sites that are mutated in these datasets and provide experimentally-derived evidence that a key and well known RBP is mutated in cancer. Finally, we provide the community with an online searchable database (http://PTM-RBP-ATLAS.igb.uci.edu) where this data can be easily gathered by the community for inspection to help with their research programs. Overall, we envision the data generated herein will serve as a powerful analysis for the growing labs interested in understanding how RBPs control the fate of RNA molecules.

## MATERIALS AND METHODS

### Aggregation of PTM sites, RNA-binding proteins, and protein–protein interactions

Known sites of post-translational modifications in human proteins were downloaded from 13 public PTM databases covering a variety of PTM types: dbSNO, HPRD, MeMo, OGlycBase, PHOSIDA, PhosphoELM, Phosphositeplus, PTM_SD, RedoxDB, SwissPalm, Swiss-Prot, SysPTM and UbiProt­ (21–33). Only experimentally determined PTMs were used; computational predictions were excluded. Additional PTM datasets not present in these databases were identified via literature search (Supplementary Table S1). All protein identifiers were converted to UniProt identifiers, and proteins present in SwissProt (31) were retained for analysis.

A list of RNA-binding proteins was derived from three publications containing extensive lists of experimentally-determined RBPs (9,10,34,35).

Protein-protein interactions were collected from 8 publicly available databases: BIOGRID, CORUM, DIP, ELM, hu.MAP, IntAct, MINT and STRING (36–43)

### PTM site conservation analysis

PhastCons conservation scores for a multiple alignment of 99 vertebrate genomes to the human genome (phastCons100way) were obtained from the UCSC Genome Browser [citation]. For each RNA binding protein, phastCons scores from 100 random positions were selected from its exons for background in comparison to scores at PTM positions.

### eCLIP mRNA binding data

eCLIP narrowPeak files from 92 RNA-binding proteins in K562 cells was downloaded from the ENCODE website (https://www.encodeproject.org/). eCLIP peak regions overlapping genes were identified using (44) bedtools to intersect eCLIP peaks with GRCh38 Gencode v24 annotations. Hits where at 50% of the peak overlaps the gene on the correct strand in both eCLIP replicates were considered instances of the RBP binding that gene.

### Cancer mutation data

Position, consequence, distribution, and frequency data were obtained for each mutation identified in The Cancer Genome Atlas and cross-referenced with identified PTM positions.

### UV mass spec sites

RNA–protein crosslink data was retrieved from four publications (45–48). As these studies employed varying techniques with differing resolutions, we adjusted all crosslink data to a 21-position range centered on the reported crosslink range. Ranges shorter than 21 positions were expanded, and those larger were truncated. PTM sites within these crosslink ranges were identified and classified by their distance from the center of the range.

## RESULTS

### Distribution and conservation of PTMs in the human RBP-ome

**Figure 1.**
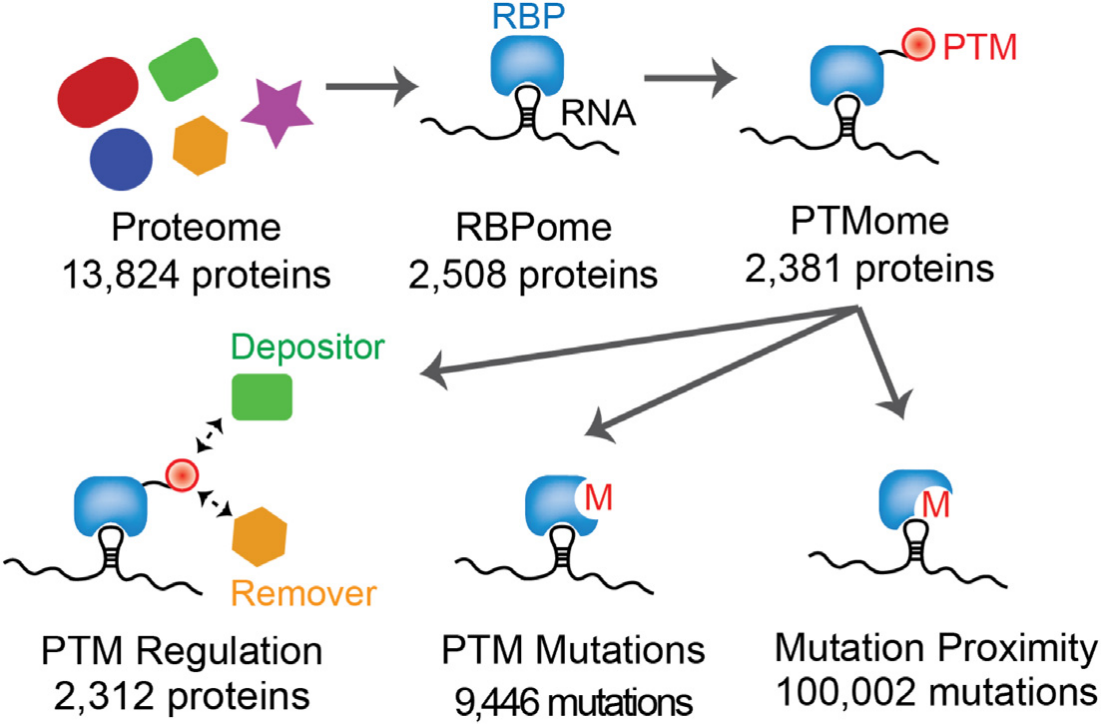
Schematic of PTM identification in the human proteome and RBPome. Clockwise from top left: count of PTM-containing proteins, total number of identified RNA binding proteins, count of RNA binding proteins with PTMs, number of cancer mutations within 10 residues of a PTM, number of cancer mutations at a PTM-bearing residue, number of modified proteins which interact with enzymes capable of depositing or removing the appropriate PTM.

Our goal in this manuscript is to better understand the landscape of PTMs on RNA binding proteins, what enzymes are responsible for their deposition, what mutations in disease relevance could alter the PTM landscape, and if PTM sites are near RNA–protein interfaces. These data points, which are important to catalog to better understand the regulation of RBPs and thus RNA biology, are represented schematically in Figure 1.

**Figure 2.**
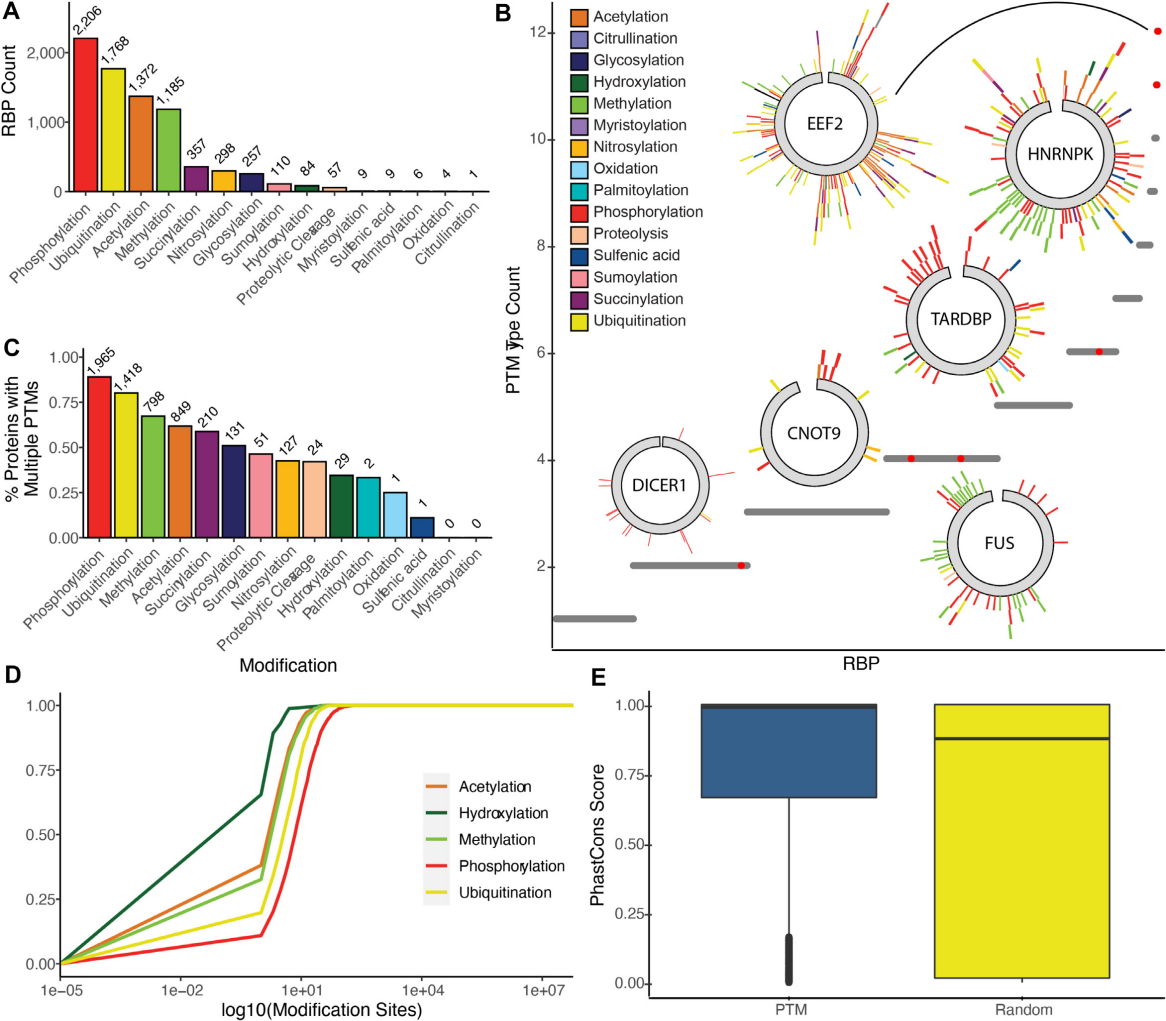
Sites of post-translational modification in RNA binding proteins. (**A**) Count of RBPs with each PTM type. (**B**) Counts of different PTM types per RBP. Location and PTM type are indicated on circular inset plots for six example RBPs with low and high PTM type counts. (**C**) Proportion of modified RBPs which contain multiple PTMs of the same type. (**D**) Cumulative distribution of modified sites per protein for five common PTM types. (**E**) Boxplot of PhastCons 100-way conservation scores for nucleotides at PTM-modified residues in RBPs versus randomly-selected positions from the same protein set. Higher scores represent more conserved positions based on genome-wide alignments of human and 99 other vertebrates.

We first worked to collate publicly available PTM sites from curated datasets and an extensive literature search. Experimentally determined PTM sites were aggregated from a set of 13 publicly available PTM databases (21–33) and an extensive literature search (Supplementary Table S1). We found modified sites were widespread, with 13 824 of the 20 239 (68%) human proteins present in SwissProt (31) containing one or more PTM sites. The union of these datasets resulted in our input set (Figure 2A)

To classify proteins as RNA-binding (the RBP-ome), we combined three existing sets of annotated RBPs from (9,10,34,35) into a non-redundant set of 2508 RBPs. Of these RBPs the vast majority are highly modified, with 2381 RBPs (95%) modified at a minimum of one site (Supplementary Table S2). This is a significantly higher proportion of modification than in the full set of human proteins (chi-squared test, *P* = 9.87 × 10^–170^). Phosphorylation was the most common modification, though acetylation, ubiquitination, and methylation were also present in over half of RBPs (Figure 2A). A total of 19 individual PTMs were significantly more common in RBPs than in all proteins, including acetylation, caspase, citrullination, glutathionylation, glycosylation, hydroxylation, methylation, myristoylation, N6-malonyllysine, nitrosylation, oxidation, palmitoylation, phosphorylation, sulfenylation, succinylation, sulfenic acid, sumoylation, ubiquitination, and Zn-Cys modifications (Chi-squared tests with Bonferroni correction, *P* < 0.05). These data highlight the potential complexity of PTM sites on RBPs.

We next worked to analyze the complexity of PTMs observed on the same RBP. Most RBPs had multiple types of PTMs (Figure 2B), with 2074 of the 2381 modified proteins having multiple modification types (87%). RBPs have significantly more PTMs per amino acid than all proteins (0.1992 versus 0.0181 modifications per amino acid, Student's *t*, *P* = 4.93 × 10^–60^). The most common set of modifications was phosphorylation, ubiquitination, acetylation, and methylation (Supplementary Figure S1), followed by phosphorylation alone or in combination with one or more of the aforementioned modifications. In addition, most RBPs (2202) have multiple instances of a PTM type (Figure 2c and d). The vast majority of phosphorylated and ubiquitinated proteins are multiply modified (88% and 81%, respectively), while multiple methylation and acetylation sites are less frequent (58% and 48%, respectively). This data may be the result of a bias in generated data as phosphorylation profiling is the most common PTM analyzed in the literature; however, the complexity of PTM classes and their dynamic nature is likely to contribute to RBP function in development and disease, as has been observed in other protein classes (13,14,49).

While most proteins have a small number of repeated modifications, there is a long tail of highly modified proteins (Figure 2D). Up to 65 acetylation, 75 methylation and 147 ubiquitination sites were observed in proteins in this dataset. Phosphorylation in particular exhibits many modifications per protein, with approximately 1% containing at least 100 phosphorylated sites. The most phosphorylated protein, serine-rich SRRM2, contained 587 sites among its 2752 residues, consistent with its known biology to be regulated by dynamic serine phosphorylation (50–52).

The presence of PTM sites is often regarded to suggest conserved function. It has been observed that PTM sites can be important for regulation of protein–protein interactions and regulatory hot spots for signaling in other classes of functional proteins (53). We worked to better understand if such high conservation would be observed in RNA binding proteins as well. Examining the conservation of modified sites, we found that within an RBP, PTM sites were more conserved across vertebrates than randomly-selected sites in 74.5% of proteins (Figure 2E). Across proteins of all types, 74.8% of modified sites were more conserved, indicating that this high level of conservation is not specific to RNA-binding proteins. For 38.6% of RBPs and 36.3% of all proteins, the mean conservation score is >0.9, indicating a high probability that they lie in elements highly conserved across species, and the higher conservation of PTM sites in RBPs is statistically significant (Kolmogorov–Smirnov test, *P* < 2e−16). This high evolutionary conservation of many PTM sites indicates they may have important functions that remain intact through evolutionary time.

Overall, our analysis of PTM sites from curated datasets and published manuscripts suggests that RBPs have highly complex experimentally observed PTM profiles, that RBPs have many different types of PTMs, and that the sites of PTM deposition are highly conserved. These resources provide a large dataset focused on RNA binding proteins for analysis by the community.

### Identification of probable PTM-depositing enzymes

**Figure 3.**
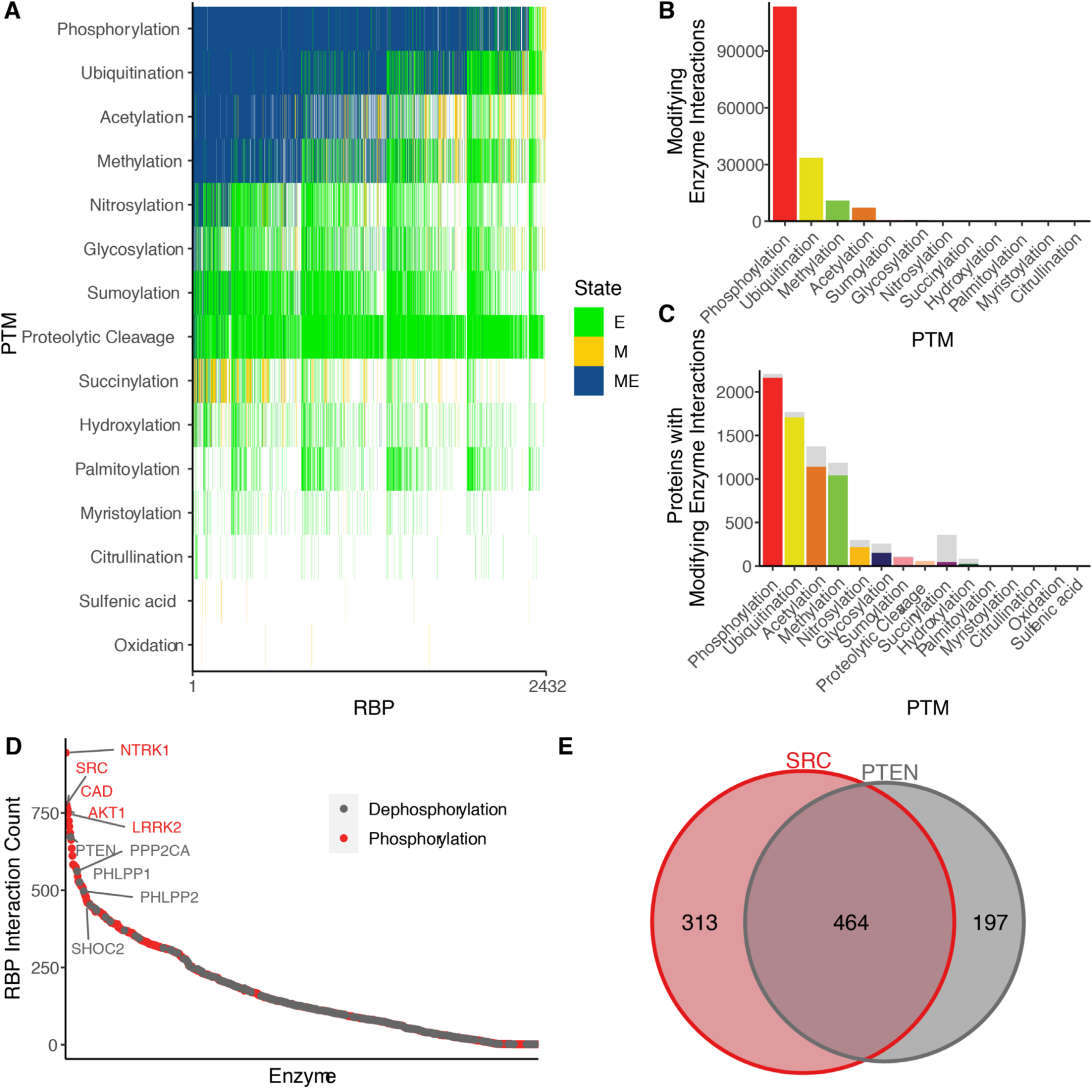
Interaction of modified RNA binding proteins with PTM-associated enzymes. (**A**) Heatmap of interaction types for each RBP and PTM type. ‘ME’ indicates that the RBP contains the indicated PTM and also interacts with an enzyme known to regulate that modification. ‘M’ indicates a protein that contains the PTM, but is not known to interact with an enzyme which regulates it. ‘E’ indicated proteins that interact with enzymes which regulate the PTM, but do not contain that PTM. (**B**) Total count of potentially modifying interactions per PTM type. Each enzyme interacting with a modified protein is counted as a separate interaction. (**C**) Count of RBPs with modifying interactions for each PTM type. Gray bars represent the total number of PTMs with the given modification. (**D**) Number of RBPs which interact with each phosphorylating or dephosphorylating enzyme. (**E**) Number of RBPs with interact with the kinase SRC and/or dephosphorylase PTEN.

PTM modifications on proteins are deposited, read and removed by distinct classes of enzymes that ultimately control PTM stoichiometry (53,54). To identify the specific enzymes with may be responsible for depositing each PTM, we cross-referenced our list of RBPs with 8 protein–protein interaction (PPI) databases (36–43). We then identified enzymes of the appropriate class which interact with RBPs carrying the corresponding PTM (from our analysis above). RBPs with known PTMs that require protein enzymes for deposition or removal, and which are known to interact with enzymes that carry out these functions, are commonly identified in these data (‘ME’; Figure 3A; Supplementary Figure S1). In addition, specific RBPs were cataloged to interact with PTM-depositing enzymatic proteins (‘E’; Figure 3A), but we did not observe these PTMs in our catalog. This may be due in part to degradation of proteins bearing certain modifications, including proteolytic cleavage and sumoylation, making these less likely to be profiled by large-scale PTM characterization efforts (55). Lastly, we identified a class of PTMs on RBPs were not associated with enzymes in protein–protein interaction databases (‘M’; Figure 3A); however, for some, this can be attributed to their nature of deposition. For example, both ‘sulfenic acid’ and ‘oxidation’ refer to oxidized forms of cysteine that are due to changes in the oxidation state of cells and do not require enzyme deposition (54,56).

Our approach identified tens of thousands of potentially modifying interactions, or cases in which a modified RBP interacts with an enzyme capable of making the same modification. Most modified proteins had at least one potentially modifying interaction; however, the percentage of proteins with such an interaction varied by PTM type (Figure 3B). For the four most common modifications (phosphorylation, ubiquitination, acetylation, and methylation), over 80% of modified RBPs have evidence for a protein–protein interaction that could control PTM stoichiometry, including >113 000 phosphorylation events. Others, like hydroxylation (29%) and succinylation (13%), had far fewer explanatory interactions. This may be due to a bias in the study of particular modifications, as kinase, methylation and acetylation signaling are well-studied for their diverse roles in regulating protein function. For many of the modifications, analysis of protein–protein interactions revealed potential enzymes that are controlling deposition. As shown in (Figure 3C), nearly all RBPs that have observed phosphorylations have associated enzymes that interact.

The large landscape of identified RBP-PTMs in which a potential enzyme pair was identified through analysis of protein–protein interactions implored us to look deeper at the identified enzyme-RBP pairs. We focused on phosphorylation as it is well studied and altered kinase activities are known to be associated with tumorigenesis (57). We ranked the interacting deposit and removal enzymes (kinase and dephosphorylase, respectively). As is shown in Figure 3D, many of the kinase and dephosphorylase enzymes interacted with a large portion of the total RBP-ome (up to 945 RBPs for kinases and 686 for dephosphorylases). Two notable proteins emerged from this analysis: PTEN and SRC kinase. Phosphatase and TENsin homolog deleted on chromosome 10 (PTEN) is a classical tumor suppressor gene, which possesses lipid and protein phosphatase activities. It has been implicated in numerous studies to control gene expression in tumorigenesis. SRC kinase is a non-receptor protein tyrosine kinase that transduces signals involved in the control of a variety of cellular processes and has been demonstrated to be important for the development, growth, progression, and metastasis of a number of different cancer types (58,59). An intersection of the PTEN-SRC target sets revealed strong overlap between the RBPs that they interact with and have been demonstrated to be phosphorylated, with 464 RBPs the binding partners of both important proteins (Figure 3E). One such RBP stuck out as a target of both enzymes: DDX5. DDX5 is notable in this case due to its interaction with oncogenic transcriptional regulators, such as nuclear factor-κβ (NF-κβ), estrogen receptor α (ERα), β-catenin, and P53, and androgen receptor. These interactions, consistent with many other protein–protein interactors could be regulated by PTMs and their deposition/removal enzymes, of which many have been observed but are not fully studied. Furthermore, DDX5 has been described as a potential as a general cancer target due to its many roles described above (60,61). Overall, our analysis of PTMs on RBPs, and the proteins that are likely responsible for their regulation is an important hypothesis generating analysis to provide a framework to the community.

### Mutations in cancer at RBP PTM sites

**Figure 4.**
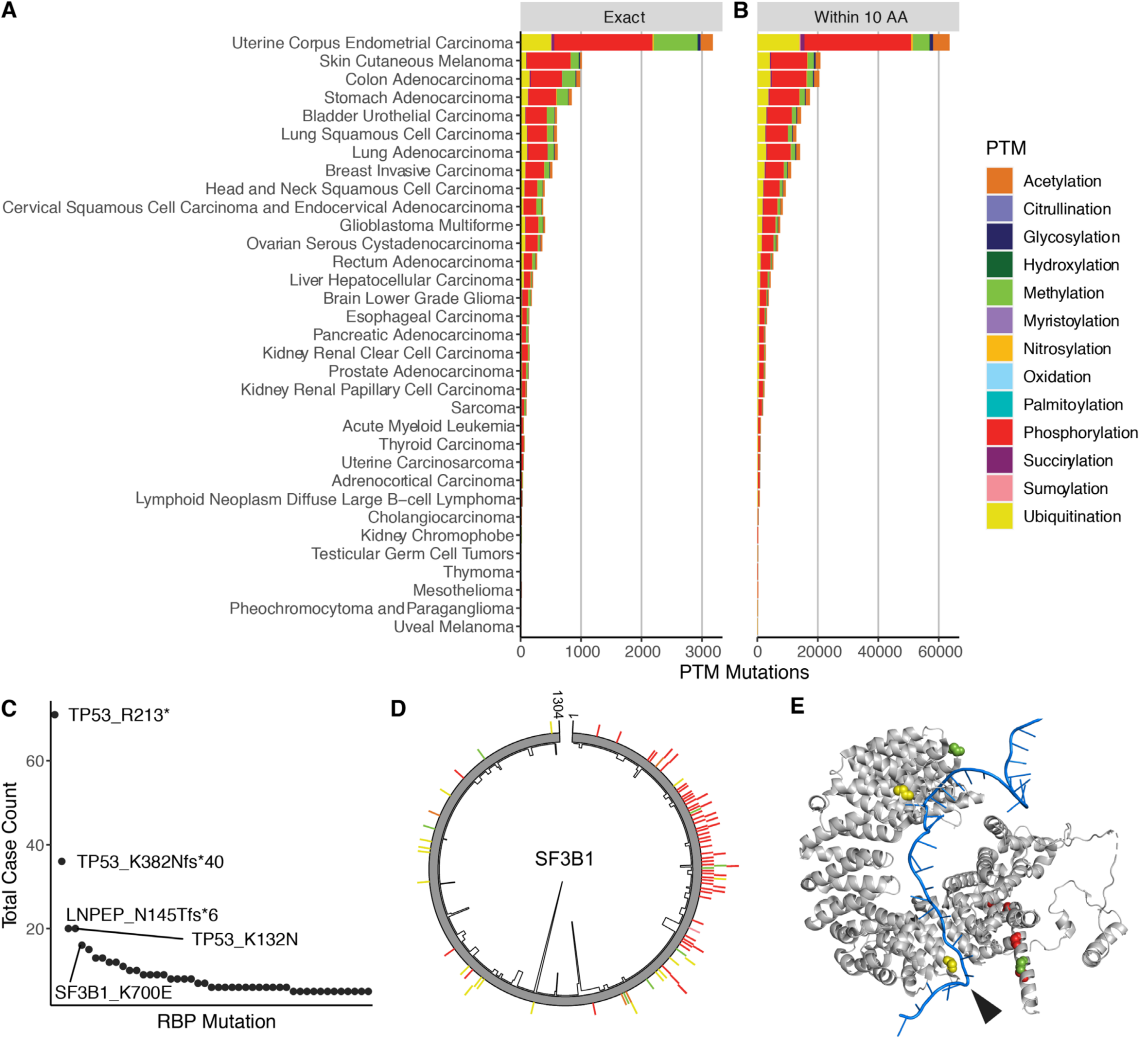
Cancer-related mutations at PTM sites. (**A, B**) Count of mutations at (**A**) or within 10 residues (**B**) of PTM sites. (**C**) Count of cancer cases with each PTM site mutation. (**D**) PTMs and mutation frequencies in the splicing protein SF3B1. The inner black line shows per-position mutation frequency; the outer colored bars show PTM types and locations. (**E**) Structure of SF3B1 (gray) with RNA (blue). Modified positions are colored according to their PTM type. The K700 position is indicated by the black arrow.

The known importance of regulation brought about by PTM sites on proteins, and identified examples in which mutation at a site of PTM altering the function of a protein prompted us to analyze mutational profiles in PTM sites in our RBP-focused analysis. To investigate the potential role of PTMs in human disease, we intersected our PTM data with mutation data from The Cancer Genome Atlas (https://www.cancer.gov/tcga). We identified 9446 cancer mutations that coincide with the position of categorized PTM modified sites in 1727 RBPs (Supplementary Table S3) (Figure 4a). We also characterized mutations surrounding PTM sites, as the local structure of proteins is known to be critical for the recognition of deposition enzymes to control PTM stoichiometry(62). From this analysis we identified 100 002 cancer mutations that fall within 10 residues of PTM modified sites in 2285 RBPs, with a similar distribution across cancer types (Figure 4B). The most mutation-rich cancer type was endometrioid carcinomas, which are known to be very unstable and as such much richer in mutations overall (63).

Analysis of the sites of mutations along RNA binding proteins provided greater understanding of which proteins are more enriched in mutations and how diverse the mutation sites are. As illustrated in Figure 4C, the most frequently observed PTM-linked mutation was at methylated position 213 in TP53, the well-known and frequently-mutated tumor suppressor. This mutation was observed in multiple patients with several cancer types, including colon, skin, breast, stomach, and uterine cancers. Consistent with p53’s known role as the ‘guardian of the genome’, its high mutation rate is associated with many cancer types (62).

One of the most common PTM-linked mutations outside of TP53 was a mutation from lysine to glutamic acid at position 700 in SF3B1 (Figure 4C). This mutation is observed in multiple patients with breast carcinoma (0.9% frequency) and thymoma (1.6% frequency), as well as single patients with prostate adenocarcinoma, cutaneous melanoma, sarcoma, and acute myeloid leukemia. This lysine is shown to be ubiquitinated in our analysis of PTM sites. Plotting all the PTM sites and mutational frequencies across SF3B1 demonstrate that the K700E site is by far the most frequently mutated (Figure 4D). SF3B1 is involved in splicing and is the most frequently mutated splicing factor in cancer, and mutation at this position is linked to loss of splicing function (64). As such, it seems that the altered K700E ubiquitin site is likely altering ubiquitin deposition and splicing. It has been demonstrated previously that ubiquitin can control protein–protein interactions involving splicing component assembly and that alterations in ubiquitin stoichiometry can lead to defects in RNA splicing (65,66). The lysine at position 700 is in close proximity to the pre-mRNA in the complex (Figure 4E); disruption of ubiquitination by mutation to glutamic acid may influence interactions with the pre-mRNA and recognition of the branchpoint sequence, leading to increased splicing at cryptic sites (67). The analysis provided herein provides a roadmap for testing such hypotheses as it relates to PTM sites on RBPs that are mutated in disease.

### Relationship between PTM sites and the RNA–protein interface

**Figure 5.**
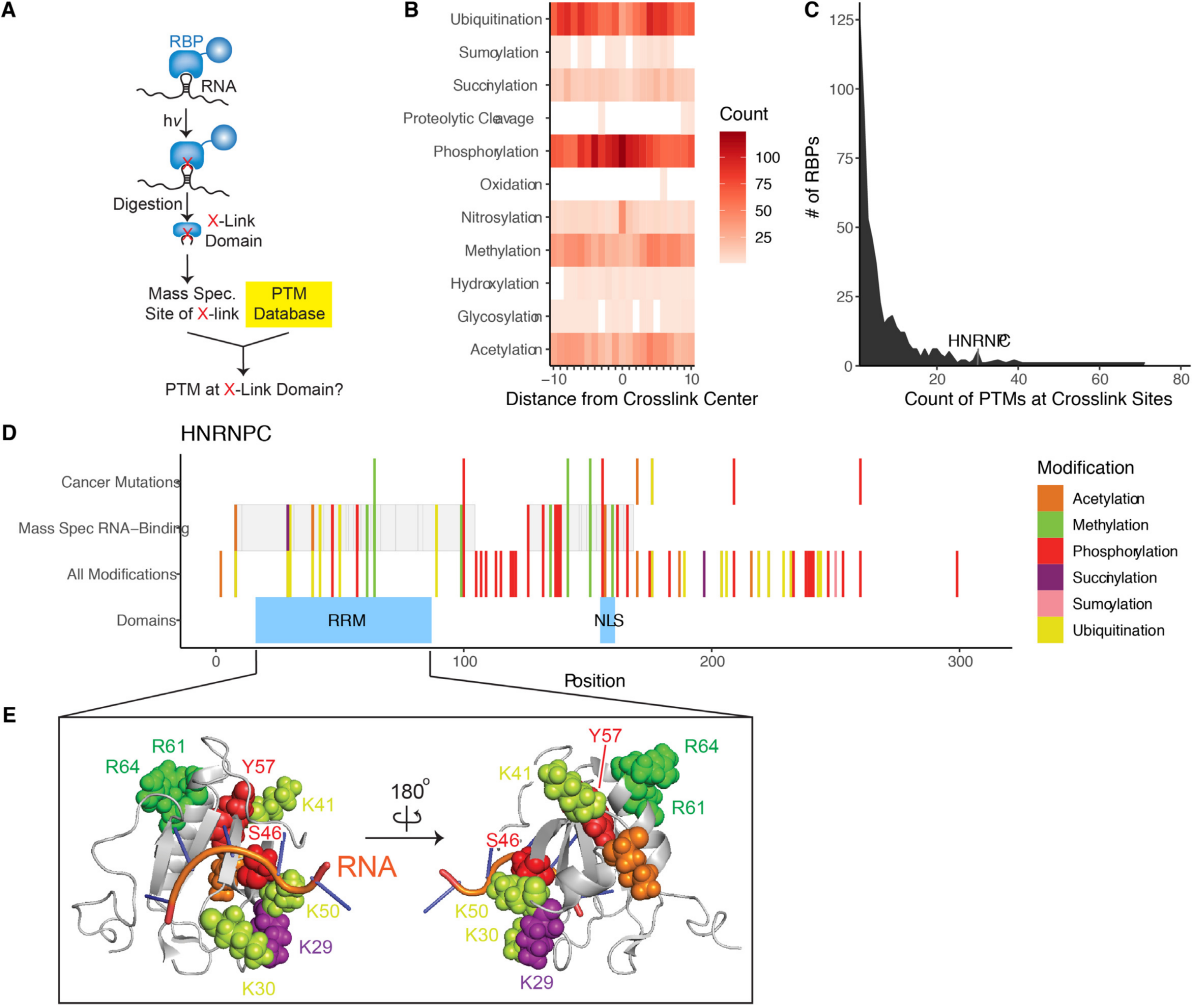
(**A**) Schematic of identification of PTMs at crosslinked domains. (**B**) Count of PTMs within 10 residues of crosslink locations. (**C**) Distribution of per-RBP PTM count at crosslink sites. (**D**) PTMs at crosslink and cancer mutation sites in HNRNPC. (**E**) Structure of hnRNP-C. Modified positions are colored according to PTM type.

RNA binding proteins function through their interaction with RNA molecules to regulate RNA structure and function. The presence of thousands of PTM sites prompted us to make headway in understanding the relationship between RNA binding and PTM occupancy in protein sequence space. There are now many published reports cataloging the cross-linked sites between RNAs and proteins through UV-mediated crosslinking. These reports provide amino-acid resolution of the RNA–protein interface (Figure 5A) (45,47,68). We sought to intersect these datasets with our PTM database to better understand the potential relationship between RNA binding and PTM deposition.

We first calculated the frequency of intersection between PTM sites and RBP–RNA crosslink peptides. In this scenario, peptides crosslinking to RNA in cells would also have PTM sites within their peptide range. In most cases PTM sites did not overlap with crosslink sites, but in rare instances proteins had a substantial amount of PTM sites that corresponded to crosslink peptide sequences (Figure 5C) (45–48). Using this data, we worked to understand the primary sequence resolution of the interactions and PTM sites, counting the number of PTMs that clustered near the crosslink peptide sites. CLIP crosslinking protocols rely on reactivity of nucleic acids and residues to form UV crosslinks; however, not all are equally reactive (69). Specific residues including lys, cys, tyr, phe and trp are more reactive for UV crosslinking, and are also subject to posttranslational modification. In cases where the reactive residue is modified, the modification could potentially interfere with the crosslinking reaction. To avoid this potential source of bias, we additionally examined PTMs up to 10 positions away from a crosslink site. As shown in Figure 5b, some PTMs had observed depositions near the center of the crosslink site, suggesting that they may be near the RNA interface of RNA binding proteins. Across all PTMs, there is an inverse relationship between distance from the nearest crosslink site and PTM frequency (Spearman's rho = –0.84, *P* < 2e–16). For individual PTMs, each had a negative association between frequency and distance from crosslink site, which was significant for 16/33 PTMs (Supplementary Table S4). The strongest association was observed for phosphorylation (rho = –0.83), followed by acetylation, ubiquitination, and methylation (rho = –0.65, –0.64 and –0.62 respectively). This observation is very exciting as the accounts of direct competition between PTM deposition and RNA binding are scarce, despite reports that PTM modifications can alter protein structure when deposited at sites other than the direct RNA–protein interface (70). Further, some types of RNA-binding proteins have been found to be intrinsically disordered, particularly in non-RNA-binding regions, and these disordered regions contain more PTM sites (71) and as such these interactions could serve as important relationships between PTMs and protein/RNA regulation to be further explored.

Inspection of the proteins with higher overlap revealed central RBPs that control numerous RNA processing functions. In particular, hnRNP proteins have a significant amount of cross link sites that are proximal to PTM sites (Supplementary Table S5). The mean distance from a PTM to the nearest crosslink site is significantly shorter in hnRNPs (36 residues) versus all other RBPs (268.4 residues; *t*-test *P*-value < 2.2e–16). We worked to analyze PTM sites at RNA–protein crosslinks from available crystal structures in the PDB. Among those, hnRNP-C stood out due to the high number of PTMs and those that intersect with cross link peptides from RNA–protein crosslinking experiments (Figure 5C). In total hnRNP-C has 63 PTM sites and 28 of them fall within the annotated RNA recognition domain (Figure 5D). hnRNP-C is a centralized RNA binding protein as part of a larger class of ubiquitously expressed heterogeneous nuclear ribonucleoproteins (hnRNPs). hnRNP-C has been demonstrated to be associated with associated with pre-mRNAs in the nucleus and appear to influence pre-mRNA processing and polyA tailing (72,73). Inspection of the crystal structure overlaid with the PTM sites we observed diverse PTMs surrounding the RNA interface of the RNA recognition motif (RRM). One notable site of PTM mutation in cancers at the RRM motif is arginine 64 (R64; Figure 5D). Arginines are known to be sites of methylation on hnRNPs; this methylation controls their nuclear export (74) and can alter their ability to bind to nucleic acids by altering arginine charge. In addition, hnRNPs contain about 65% of the total methylated arginine found in the cell nucleus(75). The interface of methylated sites on hnRNPs may be a central hub of advanced regulation. Overall, our analysis provides a better understanding of the PTM landscape and how this intersects with the RNA–RBP interface. Further inspection of individual PTM sites and their mutations in cancer should prove valuable for analysis of specific PTMs that may contribute to RBP function, RNA regulation, and potentially oncogenesis.

### An online database for access to the RBP PTM atlas

To provide the community with a working database for the ease of access to the data analyses herein, we have hosted an online database for the PTM atlas. We created a MySQL database and imported the data corresponding to 2436 RNA-binding proteins (RBPs) together with the corresponding information about 47 types of post-translational modification (PTMs). In total, there are 280 708 entries in the database, and each entry contains information about the RBP’s Uniprot ID, gene name, position, PMID, Ssource, Amino Acid (AA), modification type, cell line, sample type, body site, and disease state. The database is publicly searchable by RBP Uniprot ID or by gene name and corresponding PTM type through the web server located at: http://PTM-RBP-ATLAS.igb.uci.edu/.

## DISCUSSION

RNA biology is tightly controlled through its interactions with RNA binding proteins. Chemical modifications, termed posttranslational modifications, can control protein function. Herein we provide the community with the first global analysis of PTM marks on RNA binding proteins. Our analysis, using experimentally-determined RNA binding proteins and PTM databases reveals that RBPs are modified by a wide variety of PTMs, in all totaling 69 658 distinct modifications. We also demonstrate that the sites of PTM deposition are conserved, with many RNA binding proteins have a high level of diversity of their cataloged PTM modification types.

We have attempted to create the broadest possible sets of data for PTMs and PPIs; however, sources of bias still exist. Our data are derived from existing literature, and PTMs are not studied evenly—phosphorylation is the focus of far more studies than other PTMs, and just four modifications (phosphorylation, acetylation, ubiquitination and methylation) make up the bulk of the available data. Less-studied PTMs may not have enough data to draw significant conclusions observed with more common modifications. For PPIs, different methodologies may miss certain interactions, including transient interactions with substrates. We included eight PPI databases, which include both high-throughput and more focused experiments, using both physical and genetic techniques, to collect the most complete set of interactions possible; however, it is possible that difficult to detect interactions are underrepresented in our interaction data.

Intersection of protein–protein interaction data onto RBP modifications reveals complexity of PTM-depositing enzymes and those that could be responsible for PTM removal. Focusing on phosphorylation, an analysis of overlapping protein–protein interactions suggests master regulators that could be controlling PTM deposition and removal. This analysis highlights the potential of such enzymes, which have known roles in regulating cancer phenotypes, at center stage, thus likely controlling signaling through RBP function. Two enzymes highlighted, PTEN and SRC kinase, are known to be critically important in controlling oncogenic phenotypes, but there has yet to be a connection in their role as master regulators of RBP function and as such the potential for re-wiring the transcriptome. Our database provides a framework for generating hypotheses about potential communication between PTM regulation and RBP function ripe for testing.

We also analyzed the relationship between PTM deposition sites and mutations identified in The Cancer Genome Atlas. Our analysis revealed that many of the catalogued RBPs contain mutations, with some familiar nucleic acid binding proteins such as p53 dominating the landscape, but further analysis revealed lesser-known yet likely important mutations. For example, the SF3B1 gene encodes part of the U2 snRNP portion of the spliceosome B complex. Consistent with the literature, we observe that SF3B1 is enriched in mutations; one in particular, K700E, is now established to play an important role in regulating alternative splicing through an unknown mechanism (67). Our PTM atlas may provide a clue by virtue of the complementary analysis of PTM mass spectrometry and structural datasets demonstrating that K700 is a site of ubiquitination which may interact with the pre-mRNA undergoing splicing. A complete mechanism, further analyzed through the lens of controlling ubiquitin deposition, is sure to provide additional understanding of this modified enriched mutation site.

PTM sites control protein function, but it is unclear if proximity to the RNA–protein interface would allow them to influence RNA–protein interactions. We provide the first comparison of PTM data in the context of in-cell captured RNA–protein crosslinks. This is critical to understand if there is an overlap between the sites of PTM deposition and RNA binding. Through this analysis, the complexity of RNA recognition motifs, experimentally validated, and the relationship of PTM sites is revealed. PTM complexity and number of the hnRNP proteins, a critically important class of RBPs, comes to light. Highlighting this relationship suggests an enrichment for arginine methylation among these proteins, suggesting that neutralization of the arginine positive charge could be critically important for controlling the RBP–RNA interface. Such analysis in a fuller context of these modifications and their relationship to controlled RBP function will be invaluable to fully understand their roles.

Finally, we provide the community with an online database that is fully searchable. We hope this database, and the analyses herein provide a catalyst for experiments to fully understand the role of PTMs in regulating RBP and RNA function.

## DATA AVAILABILITY

The web interface to the database is available at: http://PTM-RBP-ATLAS.igb.uci.edu/. This website is free, open to all users and no login or password is required.

## Supplementary Material

gkac243_Supplemental_Files
