# Supplementary material for: An atlas of posttranslational modifications on RNA binding proteins
Source: Nucleic Acids Res. 2022 Apr 19;50(8):4329–39. doi: 10.1093/nar/gkac243 (PMC9071496; doi:10.1093/nar/gkac243)
Supplement: gkac243_Supplemental_Files [file gkac243_supplemental_files.zip › 220321_RBP_PTM_SUPP_REVISED.docx]

**An Atlas of Posttranslational Modifications on RNA Binding Proteins**

Whitney E. England^1^, Jingtian Wang^1^, Siwei Chen^2^, Pierre Baldi^2^, Ryan A. Flynn^3,4,*^_,_  Robert C. Spitale^*,1,5,6^

(1) Department of Pharmaceutical Sciences, University of California, Irvine. Irvine, California.

(2) School of Information and Computer Sciences, University of California, Irvine. Irvine, California.

(3) Stem Cell Program, Boston Children’s Hospital, Boston, MA.

(4) Department of Stem Cell and Regenerative Biology, Harvard University, Cambridge, MA.

(5) Department of Developmental and Cellular Biology, University of California, Irvine. Irvine, California.

(6) Department of Chemistry, University of California, Irvine. Irvine, California.

*Correspondence:

Robert C. Spitale: rspitale@uci.edu

Ryan A. Flynn: ryan.flynn@childrens.harvard.edu

**Supporting Information**

**Figure S1. An upset plot as companion to Figure 3A, showing the overall pattern/number of multiple PTM on different proteins.**
