# Supplementary material for: An atlas of posttranslational modifications on RNA binding proteins
Source: Nucleic Acids Res. 2022 Apr 19;50(8):4329–39. doi: 10.1093/nar/gkac243 (PMC9071496; doi:10.1093/nar/gkac243)
Supplement: gkac243_Supplemental_Files [file gkac243_supplemental_files.zip › 220323_RBP_PTM_MS_REVISED_TableCaptions.docx]

**Table captions for:**

**An Atlas of Posttranslational Modifications on RNA Binding Proteins**

Whitney E. England^1^, Jingtian Wang^1^, Siwei Chen^2^, Pierre Baldi^2^, Ryan A. Flynn^3,4,*^_,_  Robert C. Spitale^*,1,5,6^

Table S1: PTM datasets not present in these databases that were identified via literature search.

Table S2: Modification sites of RBPs.

Table S3: Cancer mutations that coincide with the position of categorized PTM modified sites in 1,727 RBPs.

Table S4: Association between PTM frequency and distance from RNA/protein crosslink site on RBPs.

Table S5: RNA/protein crosslink sites on RBPs.
